# Electrosurgical Septal Leaflet Liberation and Transcatheter Tricuspid Valve Replacement for Failed Postero-Septal Tricuspid Transcatheter Edge-to-Edge Repair

**DOI:** 10.1016/j.jscai.2026.105570

**Published:** 2026-08-04

**Authors:** Smitha Narayana Gowda, Ratnasari Padang, Mackram F. Eleid

**Affiliations:** Department of Cardiovascular Medicine, Mayo Clinic, Rochester, Minnesota

**Keywords:** electrosurgical leaflet laceration, transcatheter edge-to-edge repair, transcatheter tricuspid valve replacement, tricuspid valve

## Case Description

**Figure 1:**
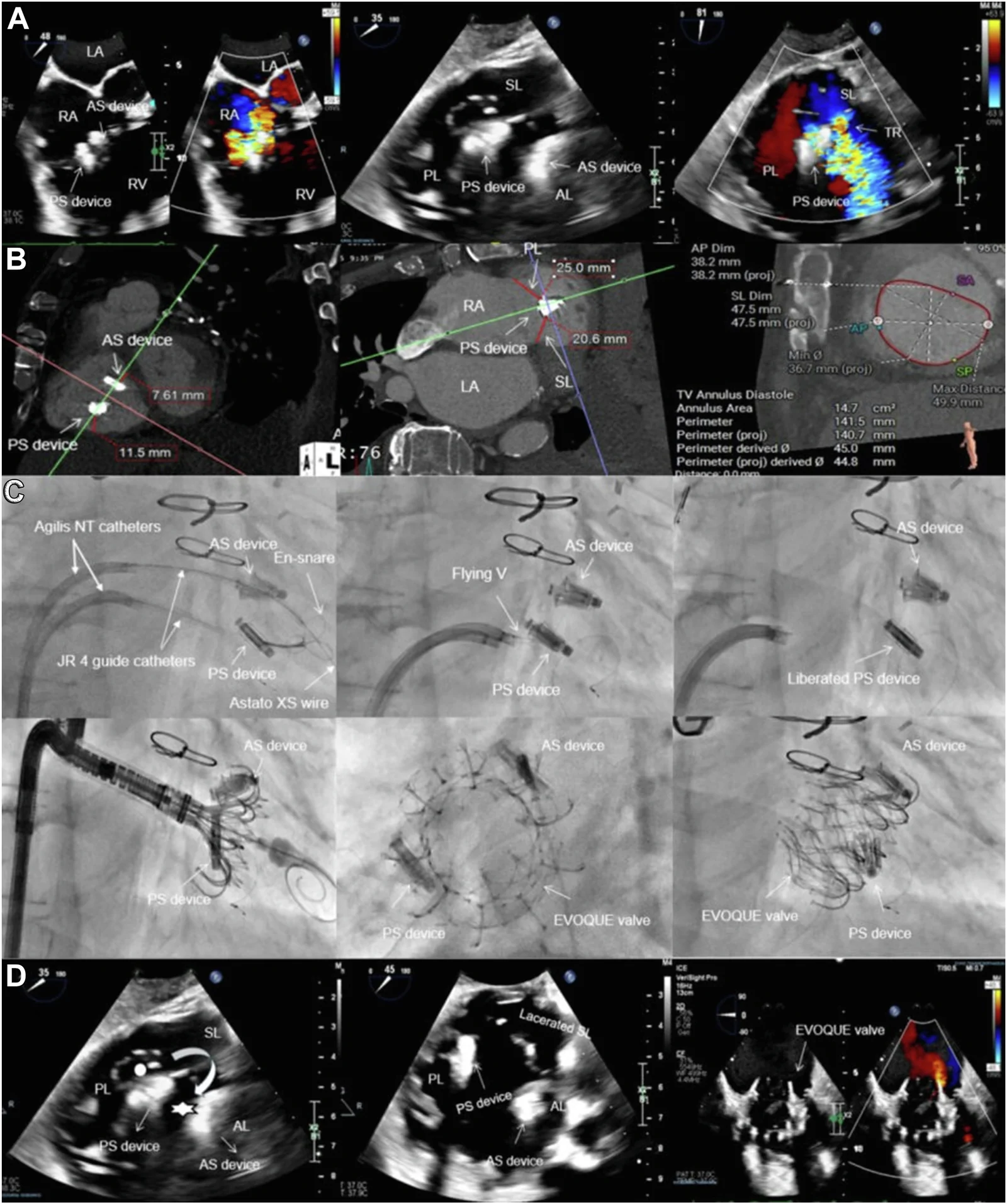
**Multimodality imaging of tricuspid TEER device laceration and TTVR.** (**A**) Transesophageal echocardiography demonstrating tricuspid TEER devices and severe TR. Mid-esophageal right ventricular (RV) inflow–outflow view showing anteroseptal (AS) and posteroseptal (PS) TEER devices with color Doppler demonstrating recurrent severe TR. Transgastric short-axis view showing PS device with poor septal leaflet tissue bridge. Severe TR from septal–posterior coaptation gap. (**B**) Cardiac CT showing device-to-annulus distance, leaflet length, and neo-annular dimensions for TTVR sizing. (**C**) Fluoroscopy of PS device laceration and TTVR. Astato wire snared with an EN Snare across the PS device. Flying-V configuration around the septal aspect of the PS device. Increased PS device mobility postlaceration. Safari wire in RV and EVOQUE valve deployment. EVOQUE anchors stabilize AS/PS devices. (**D**) Key transesophageal and intracardiac echocardiographic images of TEER device laceration and TTVR. TEE image showing the planned trajectory of electrocautery laceration (arrow), with the planned orifice for crossing (round and star). Septal leaflet-free PS device. ICE depicting EVOQUE valve with trivial residual TR. ICE, intracardiac echocardiography; TEE, transesophageal echocardiography; TEER, transcatheter edge-to-edge repair; TR, tricuspid regurgitation; TTVR, transcatheter tricuspid valve replacement.

A 70-year-old man with prior coronary artery bypass grafting, maze procedure, and De Vega tricuspid annuloplasty developed recurrent symptomatic severe tricuspid regurgitation (TR) after tricuspid transcatheter edge-to-edge repair (TEER) with 2 PASCAL Ace devices (Edwards Lifesciences) in the anteroseptal (AS) and posteroseptal (PS) positions. He presented with recurrent hospitalization for heart failure. Transesophageal echocardiography (TEE) demonstrated a stable AS device and increased mobility of the PS device due to an inadequate septal leaflet tissue bridge, with severe TR, a dominant jet arising from the coaptation gap between the septal and posterior leaflets, and no significant TR across the neo-orifice created by the AS device (Figure 1A and Supplemental Video S1). Given the prohibitive risk for redo surgery and prior tricuspid TEER, the heart team evaluated for transcatheter tricuspid valve replacement (TTVR) after PS device liberation on the septal leaflet side, due to a lack of a good tissue bridge. Cardiac computed tomography was performed to assess anatomical suitability for TTVR after PS device liberation. The AS device-to-commissure distance measured 7.6 mm, allowing the EVOQUE (Edwards Lifesciences) outer frame to conform around the prior implant and achieve adequate anchoring. Septal leaflet length was adequate for successful implantation following leaflet liberation. Neo-annular simulation after PS device liberation confirmed suitability for a 52-mm EVOQUE transcatheter tricuspid valve prosthesis (Figure 1B).

Via right femoral venous access, two 8.5F Agilis NxT catheters (Abbott) were advanced through a 26F DrySeal sheath (W. L. Gore & Associates). Two 6F JR4 catheters were crossed between the devices and through the posterior tricuspid valve orifice. An Astato XS wire (ASAHI INTECC) was snared, denuded, configured in a flying V, and positioned across the PS device at the septal leaflet-to-device interface (Supplemental Video S2). Electrocautery (45 W, 5% dextrose infusion) achieved septal leaflet laceration and PS device liberation, confirmed on TEE (Supplemental Videos S3 and S4). A 52-mm EVOQUE valve was then implanted posterior to the AS device, which acted as an anchor for the valve, stabilizing the liberated PS device with its anchors (Figure 1C, D; Supplemental Videos S5 and S6). Detailed procedural steps are provided in the Supplemental Appendix.

At 30-day follow-up, the patient had no residual heart failure symptoms (New York Heart Association functional class 1) and there was trivial residual TR on transthoracic echocardiogram. The technique of TEER device liberation after unsuccessful TEER to facilitate TTVR has recently been described by Saxon et al^1^ and represents a viable option for patients with suboptimal TEER results. Meanwhile, in cases in which the native annuli are too large for TTVR, commissural TEER can be performed to reduce neo-annulus size and enable TTVR.

In patients with prior failed tricuspid valve repair, including De Vega annuloplasty, TTVR may represent a more suitable therapeutic strategy than repeat tricuspid repair with TEER.

Further, this case demonstrates that despite partial loss of septal leaflet insertion, successful TEER device liberation with septal leaflet laceration can facilitate EVOQUE TTVR for severe TR when sufficient septal leaflet length is present.

## CRediT authorship contribution statement

**Smitha Narayana Gowda:** Writing – original draft. **Ratnasari Padang:** Writing – review & editing, Supervision. **Mackram F. Eleid:** Writing – review & editing, Supervision.

## Declaration of competing interest

The authors declared no potential conflicts of interest with respect to the research, authorship, and/or publication of this article.

## Footnotes

To access the supplementary material accompanying this article, visit the online version of the Journal of the Society for Cardiovascular Angiography & Interventions at 10.1016/j.jscai.2026.105570.
